# Intakes, Adequacy, and Biomarker Status of Iron, Folate, and Vitamin B_12_ in Māori and Non-Māori Octogenarians: Life and Living in Advanced Age: A Cohort Study in New Zealand (LiLACS NZ)

**DOI:** 10.3390/nu10081090

**Published:** 2018-08-14

**Authors:** Danika Pillay, Carol Wham, Simon Moyes, Marama Muru-Lanning, Ruth Teh, Ngaire Kerse

**Affiliations:** 1 College of Health, Massey University, Auckland 0632, New Zealand; danika.pillay@gmail.com; 2 School of Population Health, University of Auckland, Auckland 1072, New Zealand; s.moyes@auckland.ac.nz (S.M.); r.teh@auckland.ac.nz (R.T.); n.kerse@auckland.ac.nz (N.K.); 3 James Henare Māori Research Centre, University of Auckland, Auckland 1072, New Zealand; m.murulanning@auckland.ac.nz

**Keywords:** iron, folate, vitamin B_12_, biomarkers, older adults, octogenarians, LiLACS NZ

## Abstract

Advanced-age adults may be at risk of iron, folate, and vitamin B_12_ deficiency due to low food intake and poor absorption. This study aimed to investigate the intake and adequacy of iron, folate, and vitamin B_12_ and their relationship with respective biomarker status. Face-to-face interviews with 216 Māori and 362 non-Māori included a detailed dietary assessment using 2 × 24-h multiple pass recalls. Serum ferritin, serum iron, total iron binding capacity, transferrin saturation, red blood cell folate, serum folate, serum vitamin B_12_ and hemoglobin were available at baseline. Regression techniques were used to estimate the association between dietary intake and biomarkers. The Estimated Average Requirement (EAR) was met by most participants (>88%) for dietary iron and vitamin B_12_ (>74%) but less than half (>42%) for folate. Increased dietary folate intake was associated with increased red blood cell (RBC) folate for Māori (*p* = 0.001), non-Māori (*p* = 0.014) and serum folate for Māori (*p* < 0.001). Folate intake >215 µg/day was associated with reduced risk of deficiency in RBC folate for Māori (*p* = 0.001). Strategies are needed to optimize the intake and bioavailability of foods rich in folate. There were no significant associations between dietary iron and vitamin B_12_ intake and their respective biomarkers, serum iron and serum vitamin B_12_.

## 1. Introduction

The population of adults aged 85+ are expected to increase six-fold by 2068 [1] and in the coming years the older Māori population will increase at a rate higher than non-Māori [2]. The demographic shift may place increased burden on both older adults and the healthcare system. Adequate energy and nutrient intake is essential to prevent malnutrition among older adults, particularly in older Māori who are more likely to be at high nutritional risk compared to non-Māori [3,4]. Iron, folate, and vitamin B_12_ are largely intertwined in the production of red blood cells (RBC) and are the most common nutrient deficiencies associated with anemia [5,6]. Deficiency in these nutrients have also been implicated in impaired cognition and depression [7,8], increased risk of falls [9], decreased quality of life and increased risk of mortality in older adults [10,11,12,13]. 

The New Zealand Adult Nutrition Survey (NZANS) 2008/09 reported that adults aged 71+ had the lowest dietary intakes of iron and vitamin B_12_ compared to other age groups [14]. The prevalence of low iron among adults aged 71+ was relatively low (<2% of men and women) in the NZANS 2008/09; however, another investigation of adults in advanced age, the Leiden 85 + study, illustrated low iron stores ranging between 4–15.9% depending on the biomarker investigated [15]. Vitamin B_12_ deficiency was not reported in the NZANS 2008/09; however, it was previously found to be higher (12%) among adult New Zealanders over 65 years compared to younger age groups [16] and similar to findings from the Newcastle 85+ study in the UK where up to 17% of octogenarians were deficient in vitamin B_12_ [17]. Dietary folate intake was not examined in the NZANS 2008/09 due to unreliable methods of estimation, therefore, current dietary folate intake for older New Zealanders is not known. Folate stores, determined by red blood cell (RBC) folate, were mostly adequate in the Leiden and Newcastle 85+ cohorts (99.8–96.4%) [15,17]. 

There is a complex association between dietary intake and nutrient stores, and a dose-response relationship is complicated by several factors, including impaired gastrointestinal absorption [16,18,19,20], medications [21], cooking methods [21,22,23,24], and genetics [17,25]. Not all foods are equally bioavailable, for example hem iron derived from animal sources is more bioavailable and less affected by dietary factors compared to non-hem iron derived from plant sources and fortified foods [26,27]. Similarly, folic acid and synthetic vitamin B_12_ used in food fortification are more bioavailable and less affected by impaired age-related gastrointestinal absorption compared to naturally occurring food-folate and vitamin B_12_ [21,28]. Some studies have shown an association between increasing intakes of iron, folate, and vitamin B_12_ and their respective biomarkers in older adults [17,29,30,31]. However, such relationships have not been explored in New Zealanders of advanced age (80+) and may provide insights into understanding dietary adequacy of these nutrients. 

Therefore, the aim of this study was to investigate the intakes, dietary adequacy, and biomarker status of iron, folate, and vitamin B_12_, and to determine whether there is a correlation between the respective biomarkers and dietary intakes of these nutrients.

## 2. Materials and Methods 

### 2.1. Life and Living in Advanced Age: A Cohort Study in New Zealand (LiLACS NZ)

Te Puāwaitanga o Ngā Tapuwae Kia ora Tonu, Life and Living in Advanced Age: A Cohort Study in New Zealand (LiLACS NZ) is a population-based longitudinal cohort study of Māori and non-Māori in advanced age. Participants eligible for enrolment into LiLACS NZ lived in a defined geographic region of the Bay of Plenty and were born between 1 January 1925 and 31 December 1925 for non-Māori (85 years) and those born between 1920 and 1930 for Māori (80–90 years). Details of recruitment and measures have been described elsewhere [32,33]. Ethics approval for LiLACS NZ was granted by the Northern X Regional Ethics Committee (NXT 09/09/088) in December 2009 and written informed consent was obtained from all participants for each stage of the study [33]. 

At baseline, participants completed a comprehensive questionnaire, conducted by trained interviewers, and a health assessment conducted by a trained nurse. Participant age, demographic, physical and health characteristics, were established at baseline and updated if necessary during the 12-month follow-up. Medication and supplement usage established at baseline were used. For those who did not wish to do the comprehensive questionnaire, a simplified version was made available [32]. Rates of participation varied for certain measures.

### 2.2. Dietary Assessment: 24-Hour Multiple Pass Recall (MPR)

**Figure 1:**
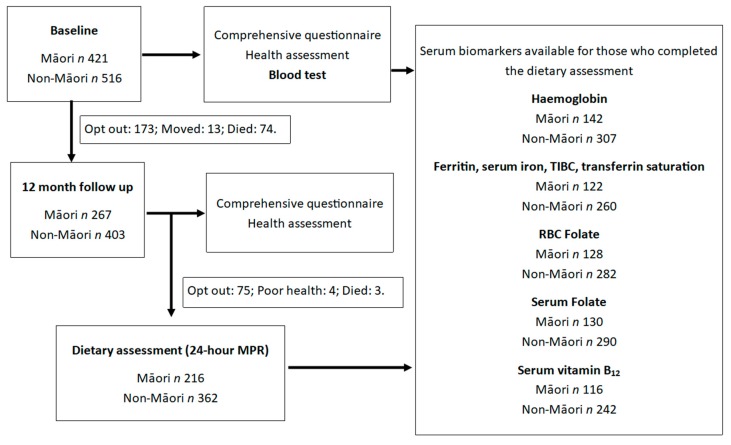
Flow chart of the number of participants who completed the 12-month dietary assessment and for whom baseline serum biomarkers were available.

Diet was assessed using two 24-h multiple pass recall (24-h MPR) completed on separate days of the week by trained interviewers (Figure 1). This method was validated for use in octogenarians as part of the Newcastle 85+ study and yielded more accurate estimates of energy and nutrient intake when compared with a food frequency questionnaire (FFQ) [34]. Where possible, the weight of food was recorded from food packages or food labels or estimated from household measuring utensils. A “Photographic Atlas of Food Portion Sizes” used in the Newcastle 85+ 24-h MPR assessments [34] was also adapted for use in the LiLACS NZ population [35] and used when portion sizes could not be ascertained from the participant, packaging, or nutrition labels. The information collected from this assessment was analyzed using FOODfiles (The New Zealand Institute for Plant and Food Research Limited and the Ministry of Health, New Zealand, 2010), an electronic subset of data from the New Zealand Food Composition Database [36].

Iron, folate, and vitamin B_12_ intakes were assessed and dietary inadequacies were determined using the Nutrient Reference Values for Australia and New Zealand [37]. Food items reported in the 24-h MPR were allocated to food groups based on the thirty-three food groups used in the 2008/09 NZANS to calculate sources of nutrients by the type of food. The main food sources contributing to iron, folate, and vitamin B_12_ have been reported elsewhere [38]. Due to the wide range of supplements used containing iron, folate, and vitamin B_12_, supplement usage was characterized as used or not used.

### 2.3. Nutritional Biomarkers

Blood samples were collected at baseline after an overnight fast. Forty mL samples were collected between 7:30 a.m. and 10:30 a.m. from the antecubital fossa vein. Blood analyzed for serum ferritin, serum iron, and total iron binding capacity were collected in plain tubes. Blood analyzed for hemoglobin, RBC folate, serum folate and serum vitamin B_12_ were collected in ethylene diamine tetraacetic acid (EDTA) tubes. The samples were centrifuged locally within 3–4 h of collection and sent immediately to the Tauranga PathLab where hemoglobin was tested within 6 hours of collection and RBC folate, serum folate and serum vitamin B_12_ were tested within 5 days. The rest of the samples were stored at −20 °C until transported to Auckland University where they were stored securely at −80 °C until the remaining tests were conducted at LabPlus. Serum folate, RBC folate, and serum vitamin B_12_ were determined using chemiluminescence (UniCel DxI 800 Immunoassay System, Beckman Coulter, Inc., Chaska, MN, USA). Hemoglobin was determined using photometric measurement (UniCel DxH 800 Coulter Cellular Analysis System, Beckman Coulter, Inc. Chaska, MN, USA). Serum iron and unsaturated iron binding capacity (UIBC) was determined using colorimetric assay (Cobas 8000, module c702, Roche Diagnostics, Manheim, Germany) and serum ferritin determined by chemiluminescence (Cobas 8000, module c602, Roche Diagnostics, Manheim, Germany). Total iron binding capacity was calculated from the addition of the serum iron concentration to UIBC.

Serum ferritin (µg/L), serum iron (µmol/L), transferrin saturation (%), and total iron binding capacity (TIBC) (µmol/L) were used to assess iron status. The cut-off for serum ferritin was defined as <12 µg/L, serum iron <10 µmol/L and TIBC >71 µmol/L [39,40]. Transferrin saturation was calculated using the equation ((serum iron / TIBC) × 100) and a cut-off <15% was used [39,40]. Serum vitamin B_12_ (pmol/L) was used to assess vitamin B_12_ status and the cut-off defined as <148 pmol/L [41]. RBC folate (nmol/L) and serum folate (nmol/L) were used to assess folate status. Cut-offs for RBC folate was classified as <317 nmol/L and serum folate <6.7 nmol/L [41,42]. Hemoglobin (g/L) was assessed to determine possible anemia which was defined as <125 g/L for men and <118 g/L for women based on National Health and Nutrition Examination Survey (NHANES) III data [43].

### 2.4. Statistical Analysis

Most statistical analyses were performed using IBM SPSS statistics package version 23 (SPSS Inc., Chicago, IL, USA). Basic descriptive analyses were completed for the number of participants, gender, ethnicity, medication usage, and dietary supplement usage for participants who had completed the 24-h MPR. Kolmogorov-Smirnov and Shapiro-Wilk tests were used to assess age, dietary intake of energy, iron, folate, and vitamin B_12_ as well as biomarkers (serum ferritin, serum iron, TIBC, Tf-saturation, RBC folate, serum folate, vitamin B_12_, and hemoglobin) for normality. Non-normally distributed data were log-transformed and retested for normality. Log-transformed normal data was expressed as the geometric mean (95th% confidence interval). Non-normally distributed variables were expressed as a median (25th, 75th percentiles). Mann-Whitney *U* tests and independent *T*-tests were used to determine ethnic and gender differences in Māori and non-Māori and men and women for energy intake, iron, folate, and vitamin B_12_ and all biomarkers.

Binary logistic regression was used to determine the odds ratio (likelihood) of deficiency in biomarkers according to quartiles of dietary intake when controlling for age, gender, and energy intake. Additional factors were added to the model based on the nutrient investigated. Estimated differences from the mean were calculated using generalized linear models controlling for age, gender, energy, and supplement intake. Models were produced by SAS 9.3 (SAS Institute Inc., Cary, NC, USA). A *p*-value < 0.05 was considered to be statistically significant and all tests were two-tailed.

## 3. Results

A total of 421 Māori and 516 non-Māori were enrolled into the study at baseline and, at 12-month follow-up, a dietary assessment was completed by a total of 216 Māori and 362 non-Māori. Those who completed the dietary assessment did not differ from those who did not with respect to living arrangements, sex, age, or depression status [32]. Serum biomarkers were not available for all participants who completed the dietary assessment and availability differed between the blood markers. Of those who completed the dietary assessment, serum biomarkers were available for hemoglobin (142 Māori and 307 non-Māori); ferritin, serum iron, TIBC and transferrin saturation (Tf-sat) (122 Māori and 260 non-Māori); RBC folate (128 Māori and 282 non-Māori); serum folate (130 Māori and 290 non-Māori); and serum vitamin B_12_ (116 Māori and 242 non-Māori) (Figure 1). An overview of the participant demographic, physical, and health characteristics and use of dietary supplements by ethnicity and gender is provided in Table 1.

### 3.1. Māori Participants

There were 43% Māori men, median age 82 (81, 85) years and 57% Māori women, median age of 84 (81, 86) years. At baseline, most Māori men (32%) and Māori women (31%) used between 4–6 medications proton pump inhibitors were used by over a third of Māori men (32%) and over a quarter of Māori women (26%). More Māori women (28%) reported consuming dietary supplements such as vitamins/minerals or multivitamins/minerals compared to Māori men (22%).

### 3.2. Non-Māori Participants

Of non-Māori, 48% were men, median age 86 (85, 86) years and 52% were women, median age 86 (85, 86) years. At baseline, most non-Māori men (35%) and non-Māori women (32%) used between 4–6 medications and a large proportion of non-Māori men (29%) and non-Māori women (36%) consumed at least one type of proton pump inhibitor. Supplements were used by 40% of all non-Māori participants with non-Māori women (49%) being the highest consumers of dietary supplements.

### 3.3. Dietary Intakes and Adequacy of Iron, Folate and Vitamin B_12_

Table 2 provides details on intake of energy, iron, folate and vitamin B_12_ in Māori and non-Māori. Overall, energy intake was significantly higher for Māori men (7.5 MJ/day) compared to Māori women (6.0 MJ/day) (*p* < 0.001). Energy intake was also significantly higher for non-Māori men (7.9MJ/day) compared to non-Māori women (6.3 MJ/day) (*p* < 0.001). Folate intake per MJ was significantly higher for Māori women (44.37 µg/MJ) compared to Māori men (36.78 µg/MJ) (*p* = 0.019). Intake of iron and vitamin B_12_ per MJ of energy did not differ significantly between men and women within their respective ethnic group. Overall, Māori consumed significantly less energy (6.4 MJ/day) compared to non-Māori (7.1 MJ/day) (*p* = 0.001) but had a significantly higher intake of vitamin B_12_ per MJ of energy (0.46 µg/MJ) compared to non-Māori (0.43 µg/MJ) (*p* = 0.038). Intake of iron and folate per MJ of energy did not differ significantly between Māori and non-Māori. 

The adequacy of dietary intake in relation to Nutrient Reference Values, (NRV’s) are also reported in Table 2. A small proportion of Māori men (12%) and women (11%) and non-Māori men (2%) and women (8%) did not meet the Estimated Average Requirement (EAR) for iron intake. A larger proportion of Māori men (58%), Māori women (58%), non-Māori men (43%) and non-Māori women (59%) did not meet the EAR for folate. Over a third of Māori women (37%) and non-Māori women (30%) did not meet the EAR for vitamin B_12_, compared with 13% of Māori men and 12% of non-Māori men. 

### 3.4. Biomarker Status

Biomarkers and the relation to recognized cut-offs for iron, folate, and vitamin B_12_ are shown in Table 2. Overall, ferritin concentrations were significantly higher in Māori (188 (94, 340)) compared to non-Māori (122 (77, 224)) (*p* = 0.002). Similarly, serum vitamin B_12_ concentrations were significantly higher in Māori (261, (95% CI = 227, 299)) than in non-Māori (222 (95% CI = 207, 239)) (*p* = 0.026). 

Hemoglobin levels were below the gender-specific reference range for ≤9% of all women and ≤16% of all men. Only one percent of non-Māori were below the cut-off for serum ferritin. Few participants had low serum iron (<5%) and low transferrin saturation (<6%). Few participants exceeded the cut-off for TIBC (<7%). Up to a third of men and women were found to have a RBC folate level below the reference range (<317 nmol/L). Serum folate was below the reference range for only 2% of Māori men and women (6.7 nmol/L). Serum vitamin B_12_ deficiency was seen in ≤11% of all participants. 

### 3.5. Top Food Contributors for Iron, Folate and Vitamin B_12_ Intake

The top food sources contributing to dietary intakes of iron, folate and vitamin B_12_ for Māori and non-Māori are reported in Table 3. Food sources contributing to the majority of iron intake for Māori and non-Māori men included cereals (18%; 19%), bread (12%; 14%), and beef and veal (8%; 9%). For Māori and non-Māori women, the top three sources of iron included cereals (17%; 15%), bread (15%; 15%) and vegetables (8%; 9%). 

For dietary folate intake main food sources for Māori men and women, respectively, were cereals (20%; 18%), vegetables (15%; 16%) and bread (14%; 16%). Major sources for non-Māori men were cereals (19%), bread (16%) and vegetables (14%). Non-Māori women had most of their dietary folate from vegetables (16%), followed by bread (15%) and cereals (14%).

Greatest food source contributors to vitamin B_12_ intake for Māori men and women, respectively, were milk (20%; 24%), fish and seafood (16%; 16%), and beef and veal (17%; 15%). The majority of vitamin B_12_ for non-Māori men and women, respectively, came from milk (23%; 24%), fish and seafood (12%; 9%), and beef and veal (21%; 16%).

A comprehensive analysis of food sources contributing to dietary intake of iron, folate, and vitamin B_12_ has been reported elsewhere [38]

### 3.6. Association between Dietary Intake and Biomarker Status

**Figure 2:**
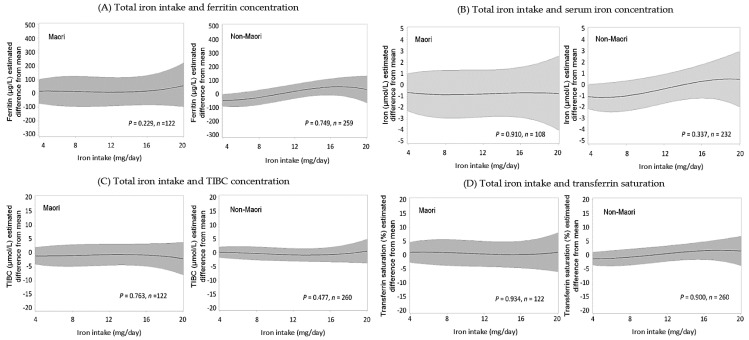

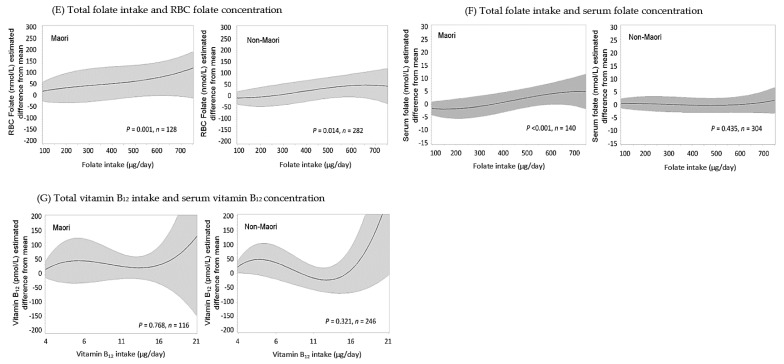
Estimated the difference from the mean (and 95% CI) of biomarkers for iron, folate, and vitamin B_12_ according to total dietary intakes of the respective nutrient for Māori and non-Māori. Estimates were calculated using generalized linear models controlling for age, gender, energy intake and supplement usage. *p*-values are from the corresponding model on the ability of dietary intake to predict biomarker concentrations. (**A**) Association between dietary iron intake (mg/day) and ferritin (µg/L). (**B**) Association between dietary iron intake (mg/day) and serum iron (µmol/L). (**C**) Association between dietary iron intake (mg/day) and total iron binding capacity (µmol/L). (**D**) Association between dietary iron intake (mg/day) and transferrin saturation (%). Estimated the difference from the mean (and 95% CI) of biomarkers for iron, folate, and vitamin B_12_ according to total dietary intakes of the respective nutrient for Māori and non-Māori. Estimates were calculated using generalized linear models controlling for age, gender, energy intake and supplement usage. *p*-values are from the corresponding model on the ability of dietary intake to predict biomarker concentrations. (**E**) Association between dietary folate intake (µg/day) and RBC folate concentrations (nmol/L). (**F**) Association between dietary folate intake (µg/day) and serum folate concentrations (nmol/L). (**G**) Association between dietary vitamin B_12_ intake (µg/day) and serum vitamin B_12_ concentrations (pmol/L).

For Māori, dietary iron intake was not significantly associated with serum ferritin (*p* = 0.229) or serum iron levels (*p* = 0.910) (Figure 2). For non-Māori, a positive but non-statistically significant trend was observed for serum ferritin and serum iron concentration with increasing iron intake. For both Māori and non-Māori iron intake was not a significant predictor of TIBC or transferrin saturation. 

Controlling for age, gender, intake of energy, supplements and folic acid, dietary folate intake was a significant predictor of RBC folate levels for Māori (*p* = 0.001) and non-Māori (*p* = 0.014) (Figure 2). Māori and non-Māori participants were more likely to have a higher RBC folate concentration if their folate intake was higher. Dietary folate intake was a significant predictor of serum folate concentration for Māori (*p* < 0.001).

Models for Māori and non-Māori showed a trend towards increasing serum vitamin B_12_ concentration with an increase in vitamin B_12_ intake increased but was not statistically significant. 

### 3.7. Risk of Iron, Folate or Vitamin B_12_ Deficiency by Quartiles of Dietary Intake

Table 4 shows the odds ratio and 95% CI of deficient RBC folate (<317 nmol/L) according to total folate intake quartiles. For Māori, as intake of folate increased, the odds of having low RBC folate decreased (significant at all intakes >215 µg/day). The same significant trend was found when adjusted for covariates. In the unadjusted model, non-Māori individuals with an intake >440 µg/day were significantly less likely to be deficient in RBC folate when compared to individuals with an intake <215 µg/day. However, when adjusted for gender, energy intake, nutritional supplement usage and intake of folic acid, a similar magnitude but not statistically significant trend was found. There were no significant associations between the dietary intake of iron and vitamin B_12_ and their respective biomarkers, serum iron and serum vitamin B_12_.

## 4. Discussion

Overall, for Māori and non-Māori participants dietary iron intakes and biomarkers for iron were largely adequate. Similarly, the EAR for vitamin B_12_ was met by three quarters of Māori and non-Māori participants. Māori had higher intakes of vitamin B_12_ (*p* = 0.038) and serum vitamin B_12_ (*p* = 0.026) compared to non-Māori. The EAR for dietary folate was met by less than half of Māori and non-Māori suggesting a sub-optimal intake. A higher dietary folate intake was associated with increased RBC folate for Māori (*p* = 0.001) and non-Māori (*p* = 0.014) and with increased serum folate for Māori (*p* < 0.001), suggesting that a higher intake is related to higher serum biomarker levels. Folate intake >215 µg/day was associated with a reduced risk of deficiency in RBC folate for Māori (*p* = 0.001) which suggests that increased dietary folate intake is positively associated with RBC folate. 

### 4.1. Iron

In the present study, most participants had an adequate dietary iron intake (88% Māori; 95% non-Māori met the EAR). Inadequate dietary iron intake was evident in a larger proportion of Māori (12% men; 8% women) and non-Māori (2% men; 8% women), compared to <3% of adults aged 71+ reported to have intakes below the EAR in the NZANS 2008/09 [14]. Given the dietary assessment method was similar in the NZANS 2008/09 [42], and iron intake was similar between adults aged 71+ (men, 11.4 mg/day; women, 8.9 mg/day) and participants in the present study (Māori, 9.7 mg/day; non-Māori, 10.6 mg/day), the discrepancies may arise as a result of the probability analysis used in the NZANS 2008/09 to estimate inadequate dietary intake. This method may have led to an overestimation of dietary adequacy in older adults, as opposed to the direct comparison to the EAR as used in the present study [42]. 

Only one participant had a low serum ferritin, similar to findings in the NZANS 2008/09 where less than 2% of adults aged 71+ had low ferritin [14]. As serum ferritin concentration may increase with age and inflammation, it has been proposed that a higher cut-off be used to assess deficiency [44,45,46]. Although not observed in the current study, previous reports have demonstrated a positive correlation between dietary iron intake and serum ferritin in octogenarians [29,47]. We found most participants had adequate levels of serum iron (96% Māori; 95% non-Māori), TIBC (95% Māori; 97% non-Māori) and transferrin saturation (95% Māori; 94% non-Māori). Lower dietary iron intake has previously been correlated with lower serum iron concentration and higher TIBC in this age group [48] but dietary iron intake was not significantly associated with serum ferritin, serum iron, TIBC, and transferrin saturation in the present study and increasing dietary iron intake was not significantly associated with a lesser likelihood of deficiency.

There are underlying limitations in assessment of iron biomarkers. Bioavailability may impact dietary iron status [26]. For example, it is estimated that >15% of hem iron is absorbed versus <5% of non-hem iron due to diet-related factors [27]. Inflammation may also influence ferritin, serum iron, TIBC, and transferrin saturation [39,45]; hence markers may be difficult to interpret in the absence of inflammatory status. This is particularly relevant to this age group where chronic low-grade inflammation may be present [15]. As serum iron and transferrin saturation may also be subject to diurnal variation [49], this may further explain why the iron biomarkers were unrelated to dietary intake.

As expected, men (both Māori and non-Māori) had significantly higher serum ferritin and lower TIBC concentrations compared to women. Men derived more iron from cereals, bread, and beef and veal whereas women had more iron from non-hem sources such as cereals, bread, and vegetables. Select cereals and bread have been fortified with iron; however, bioavailability may be reduced due to the presence of phytates and fiber [26,50]. Therefore, the higher intake of hem iron from beef and veal among men may explain why their iron stores were larger. This may also reflect men traditionally having higher iron stores and energy intakes compared to women [14,38,51].

### 4.2. Folate

Less than half of all participants met the EAR for folate (42% Māori; 49% non-Māori) and up to a third of participants were deficient in RBC folate (27% Māori; 31% non-Māori). Increased dietary folate intake was significantly associated with increased RBC folate concentration for both Māori and non-Māori and with increased serum folate concentrations for Māori only. As RBC folate is a marker of long-term folate status and a large proportion of participants appeared deficient, it could indicate that consumption of folate-rich foods is inadequate, given that dietary intake is a major influencer of folate biomarkers [52,53] and intake was shown to be low in this study.

Synthetic folic acid, used in the fortification of bread and cereals, is more bioavailable (~85%) than folate found naturally in foods (~50%) [21,54]. In the Newcastle 85+ study, a higher intake of cereal and cereal products was significantly and positively associated with RBC folate concentrations in octogenarians [17]. In this study, cereals and bread, subject to voluntary fortification in New Zealand, made up the majority of dietary folate intake for both Māori and non-Māori and Māori with a higher intake of folate (>215 µg/day) and were significantly less likely to be deficient in RBC folate compared to those who had intakes <215 µg/day. This trend was also observed in the Newcastle 85+ study where the likelihood of deficiency in RBC folate was more than halved when dietary intake of folate was >264 µg/day compared to dietary intakes of <157 µg/day [17].

There may be a decline in dietary intakes of folate-rich fruit and vegetables with ageing [30] because of declining oral health and poor chewing ability [55]. Furthermore, up to 80% of folate in vegetables can be destroyed through boiling [21,55]. Incomplete liberation from cellular structures in the food matrix, and other dietary components, including ascorbic acid, have also been shown to influence absorption [52,54,56]. These factors may explain the higher prevalence of inadequate dietary intake and RBC folate deficiency in the present study, compared to the 2008/09 NZANS where <3% of adults aged 71+ and Māori aged 51+ had deficient RBC folate concentrations [14]. However, it was beyond the scope of the study to establish cooking practices. Serum folate deficiency in the present study was rare (0% Māori; 2% non-Māori), similar to the findings from the 2008/09 NZANS where <2% of adults aged 71+ and Māori aged 51+ were below the cut-off (6.7 nmol/L) [14]. However, serum folate is a measure of short-term folate status and can also be affected by recent dietary intake which may explain the discrepancies between RBC folate and serum folate [57]. 

Māori women tended to have significantly higher energy-adjusted folate intake (44.37 µg/MJ), yet were more likely to be deficient in RBC folate (29%) compared to Māori men (36.78 µg/MJ; 24%). In the present study, Māori women were more likely to get their folate from fortified sources such as cereals and bread compared to cereals and vegetables for Māori men. Given that the New Zealand Food Composition Database relies on manufacturers claims regarding folic acid content of folate-fortified products, it could lead to an overestimation of dietary intake [42]. Generally, men also tended to have a higher energy intake which may further explain that despite Māori women having higher energy-adjusted folate intakes, they still had a higher prevalence of RBC folate deficiency compared to men. Regardless, food sources fortified with folic acid appear to be the most important source of folate for older adults.

### 4.3. Vitamin B_12_

A quarter of participants did not meet the EAR for vitamin B_12_ (26% Māori; 22% non-Māori), yet, serum vitamin B_12_ was mostly adequate among the study participants (93% Māori; 91% non-Māori above the cut-off: 148 pmol/L) which may reflect that vitamin B_12_ stores were largely adequate in this population. Unsurprisingly, more men met the EAR for vitamin B_12_ (87% Māori; 88% non-Māori) compared to women (63% Māori; 70% non-Māori). The results are similar to that reported in the NZANS 2008/09 where fewer women aged 71+ met the EAR (73%) compared to men (96.2%) [14]. Men tended to derive more vitamin B_12_ from sources such as beef and veal (17% Māori; 21% non-Māori) compared to women (15% Māori; 16% non-Māori) and women derived slightly more from sources lower in vitamin B_12_ such as milk (24% Māori and non-Māori) compared to men (20% Māori; 23% non-Māori). 

The vitamin B_12_ present in meat products can range anywhere between 0.7–5.2 µg/100 g depending on the animal origin, cooking method, and cut of meat, and can be up to 110 µg/100 g for animal liver whereas the vitamin B_12_ content of milk is much lower and can range between 0.2–0.4 µg/100 g [24] which may explain why more men had adequate dietary intakes compared to women. However, it has also been shown that the bioavailability of food-bound vitamin B_12_ is higher when the vitamin B_12_ content of the food is lower [24] which may explain why fewer women met the EAR compared to men, but it was not reflected in their serum vitamin B_12_ concentrations. It may indicate that a more frequent intake of sources lower in vitamin B_12_ versus one dietary source high in vitamin B_12_ is better for maintaining adequate serum levels [24].

An increased intake of vitamin B_12_ was not significantly associated with serum vitamin B_12_ status or a lesser likelihood of deficiency in serum vitamin B_12_. Several studies indicate that serum vitamin B_12_ levels tend to saturate with dietary intakes of approximately 10 µg/day and older adults with intakes >2.88 µg/day may be half as likely to be deficient in serum vitamin B_12_ compared to those who have intakes <1.87 µg/day [17,58,59]. Older adults may have decreased secretion of intrinsic factor, essential for vitamin B_12_ absorption, which may result in malabsorption of vitamin B_12_ from dietary sources [60]. In addition, stores of vitamin B_12_ can last several years before depletion which may also explain the observed lack of association between dietary intake and serum vitamin B_12_ in the present study [61].

### 4.4. Strengths and Limitations

This study provides the first detailed examination of iron, folate, and vitamin B_12_ intakes and biomarker status of Māori and non-Māori octogenarians. Robust methods of recruitment were undertaken as part of the comprehensive longitudinal study (LiLACS NZ) which led to an adequate representation of Māori and non-Māori [33]. The 24-h multiple pass recall dietary assessment tool has been validated for use in octogenarians and provides more realistic estimates of energy and nutrient intake compared to an FFQ [34]. 

Blood samples were rapidly processed after collection providing the most accurate estimates, and when blood could not be analyzed, it was securely stored at the appropriate temperature ensuring its stability until it could be processed. Blood samples were collected at baseline and dietary data was collected at the 12-month follow-up. Hence, intakes of these nutrients at the 12-month follow-up may not be accurately representative of intakes at baseline during the blood collection. Further, given that blood test results were communicated to the participants’ general practitioner, any action to correct deficiencies such as supplementation and dietary advice is not known. 

The biomarker cut-offs and EARs used in the present analysis are based on adult studies and are not specific for adults aged 80+ years hence may not represent nutrition-related health outcomes for this age group. Additionally, there is no clear universal cut-off for serum vitamin B_12_ to define deficiency although several have been proposed ranging between 148 pmol/L–250 pmol/L [41,57,60,62] therefore, if a higher cut-off had been used there may have been a higher rate of deficiency observed. Holotranscobalamin measures the active form of vitamin B_12_ and could be a more accurate diagnostic marker for metabolic vitamin B_12_ deficiency [63,64,65]. It is possible therefore that the proportion of participants identified as being deficient in vitamin B_12_ may be greatly underestimated using serum vitamin B_12_ to define vitamin B_12_ deficiency. 

In the 12-month follow-up, a third (35%) of participants were found to be potential misreporters based on an EI: BMR_est_ < 0.9 and EI: BMR_est_ > 2.0 [38] and, as this may influence some of the significant contrasts, interpretation should be made with caution. 

Participants who used supplements including vitamins/minerals and multivitamins/minerals were not excluded from the study. Therefore, dietary intake and supplement usage is intertwined, and interpretation of biomarkers should be taken with caution. Further, the generalized linear models and binary logistic regression included participants using supplements which may have skewed the results given that the bioavailability of supplemental folic acid and vitamin B_12_ is higher than that found naturally in food. The results may have differed if these participants were excluded.

## 5. Conclusions

In summary, dietary intakes and stores of iron were largely adequate among the study participants. More men met the EAR for vitamin B_12_ compared to women; however, vitamin B_12_ stores were not significantly different between genders and dietary intake did not correlate with serum vitamin B_12_. More than half of participants did not meet the EAR for folate and up to a third of participants were deficient in RBC folate. This suggests that dietary folate may be inadequate in the diets of octogenarians and food-based strategies to improve intake need to be investigated.

## Figures and Tables

**Table 1:** Participant Characteristics and Baseline Medication and Supplement Usage for Māori and Non-Māori by Gender.

Participant Characteristics		Māori			Non-Māori		
		Total	Men	Women	Total	Men	Women
Number of participants, n (%) *		216	92 (43%)	124 (57%)	362	172 (48%)	190 (52%)
Age (years) ^1^		83 (81, 85)	82 (81, 85)	84 (81, 86)	86 (85, 86)	86 (85, 86)	86 (85, 86)
Medications used ^2^	None	36 (17%)	15 (16%)	21 (17%)	14 (4%)	9 (5%)	5 (3%)
	1–3	35 (15%)	14 (15%)	21 (17%)	53 (15%)	29 (17%)	24 (13%)
	4–6	68 (31%)	29 (32%)	39 (31%)	120 (33%)	60 (35%)	60 (32%)
	7–9	44 (20%)	21 (23%)	23 (19%)	97 (27%)	40 (23%)	57 (30%)
	10+	33 (15%)	13 (14%)	20 (16%)	78 (22%)	34 (20%)	44 (23%)
Types of Medications used	Antacids	3 (1%)	3 (3%)	0	3 (1%)	1 (1%)	2 (1%)
	H_2_RAs	0	0	0	4 (1%)	1 (1%)	3 (2%)
	PPIs	61 (28%)	29 (32%)	32 (26%)	118 (33%)	50 (29%)	68 (36%)
	NSAIDs	18 (8%)	6 (7%)	11 (9%)	69 (19%)	26 (15%)	43 (23%)
Dietary supplements ^3^	None	161 (75%)	72 (78%)	89 (72%)	217 (60%)	120 (70%)	97 (51%)
	Supplements used	55 (25%)	20 (22%)	35 (28%)	145 (40%)	52 (30%)	93 (49%)

Values expressed as number (percentage) unless otherwise specified. * includes all participants who completed the 24-h MPR dietary assessment at 12-month follow-up. ^1^ Expressed as median (IQR). ^2^ including dietary supplements; ^3^ includes intake of vitamins, minerals and/or multivitamins/minerals. N.B. Missing values indicate some questionnaires were incomplete; therefore, rates of participation will vary. Abbreviations: H2RAs, Histamine-2 Receptor Antagonists; PPIs, proton pump inhibitors; NSAIDs, non-steroidal anti-inflammatory drugs.

**Table 2:** Iron, folate and B_12_: Dietary intakes and adequacy in relation to NRVs, nutritional biomarkers in relation to cut-offs, and top food contributors for Māori and non-Māori men and women who completed the dietary assessment at 12-month follow-up.

	Māori			*p*-Value ◊	Non-Māori			*p*-Value ¥	*p*-Value Ethnic *
	Total	Men	Women		Total	Men	Women		
Energy Intake (MJ) ^1^	6.4 (5.2, 8.2)	7.5 (6.1, 9.1)	6.0 (4.8, 7.2)	<0.001	7.1 (5.8, 8.7)	7.9 (6.7, 9.6)	6.3 (5.3, 7.5)	<0.001	<0.001
**Iron**									
Intake (mg/day) ^1^	9.7 (7.1, 13.1)	11.2 (7.8, 14.4)	8.9 (6.7, 11.9)	-	10.6 (8.1, 13.3)	11.6 (9.9, 14.3)	9.3 (7.1, 11.7)	-	-
Intake per 1 MJ (mg)	1.47	1.43	1.54	0.376	1.44	1.44	1.42	0.692	0.391
<EAR (n, %) ^a^	25 (12%)	11 (12%)	14 (11%)	-	18 (5%)	3 (2%)	15 (8%)	-	-
Hemoglobin (g/L) ^1^	137 (128, 144)	137 (126, 146)	134 (127, 143)	0.197	136 (126, 143)	138 (127, 144)	129 (123, 138)	<0.001	0.167
Hb <125 g/L (men); or <118 g/L (women)	15 (7%)	10 (11%)	5 (4%)	-	45 (12%)	28 (16%)	17 (9%)	-	-
Ferritin ^1^	188 (94, 340)	257 (127, 369)	172 (84, 304)	0.015	122 (77, 224)	179 (97, 361)	107 (71, 178)	<0.001	0.002
<12 (µg/L)	0	0	0		1 (1%)	1 (1%)	0		
Serum iron (µmol/L) ^1^	15 (11, 18)	14 (11, 17)	15 (12, 20)	0.544	15 (12,18)	18 (12, 19)	15 (12,17)	0.146	0.882
<10 µmol/L	8 (4%)	3 (3%)	5 (4%)	-	18 (5%)	8 (5%)	10 (5%)	-	-
TIBC (µmol/L) ^1^	57 (52, 65)	56 (51, 62)	57 (52, 67)	0.015	56 (50, 63)	52 (48, 57)	57 (53, 65)	<0.001	0.080
>71 µmol/L	10 (5%)	2 (2%)	8 (7%)	-	9 (3%)	5 (3%)	4 (2%)	-	-
Tf-sat (%) ^1^	28 (21, 34)	28 (22, 35)	28 (21, 35)	0.545	28 (22, 34)	32 (24, 39)	26 (22, 32)	0.001	0.544
<15%	10 (5%)	4 (4%)	6 (5%)	-	21 (6%)	10 (6%)	11 (6%)	-	-
**Folate**									
Intake (µg/d) ^1^	279 (191, 393)	298 (205, 391)	268 (186, 397)	-	313 (224, 447)	341 (253, 479)	290 (202, 407)	-	-
Intake per 1 MJ (µg)	42.15	36.78	44.37	0.019	44.31	42.22	45.61	0.304	0.417
<EAR (*n*, %) ^b^	125 (58%)	53 (58%)	72 (58%)	-	186 (51%)	74 (43%)	112 (59%)	-	-
Serum folate (nmol/L) ^2^	18 (16–20)	18 (16–20)	19 (17–21)	0.110	19 (17–20)	17 (14–20)	19 (16–21)	0.480	0.430
<6.7 nmol/L	0	0	0	-	6 (2%)	3 (2%)	3 (2%)	-	-
RBC folate (nmol/L) ^2^	338 (302–379)	333 (298–372)	338 (315–363)	0.635	336 (317–357)	322 (261–398)	348 (303, 401)	0.866	0.228
<317 nmol/L	58 (27%)	22 (24%)	26 (29%)	-	111 (31%)	54 (31%)	57 (30%)		-
**Vitamin B_12_**									
Intake (µg/d) ^1^	3.0 (1.9, 4.4)	3.4 (2.5, 5.1)	2.7 (1.7, 3.9)	-	3.03 (2.1, 4.2)	3.6 (2.4, 4.8)	2.6 (1.8, 3.6)	-	-
Intake per 1 MJ (µg)	0.46	0.48	0.45	0.198	0.43	0.45	0.42	0.370	0.038
<EAR (*n*, %) ^c^	55 (26%)	12 (13%)	43 (37%)	-	78 (22%)	21 (12%)	57 (30%)	-	-
Serum B_12_ (pmol/L) ^2^	261 (227–299)	212 (188–239)	229 (209–251)	0.359	222 (207–239)	253 (196–363)	265 (224–315)	0.770	0.026
<148 pmol/L	15 (7%)	6 (7%)	9 (7%)	-	34 (9%)	19 (11%)	15 (8%)	-	-

^1^ Expressed as median [IQR]; ^2^ Expressed as geometric mean and 95% confidence interval; ^a^ Based on an EAR 6 mg/d for men and 5 mg/d for women aged >70 years; ^b^ Based on EAR 320 µg/d for men and women aged >70 years; ^c^ Based on an EAR of 2.0 µg/d for men and women aged >70 years. ◊ Differences between Māori men and Māori women (Independent samples *T*-test, Mann-Whitney *U* Test (two-tailed)); ¥ Differences between non-Māori men and non- Māori women (Independent samples *T*-test, Mann-Whitney U Test); * Differences between all Māori and all non-Māori participants (Independent samples *T*-test, Mann-Whitney *U* Test). *p*-value < 0.05 considered significant. Abbreviations: MJ, mega-joules; EAR, estimated average intake; Hb, hemoglobin; TIBC, total iron binding capacity; Tf-sat, transferrin saturation; RBC, red blood cell.

**Table 3:** Top food sources contributing to dietary intakes of iron, folate and vitamin B_12_ for Māori and non-Māori men and women.

	Māori		Non-Māori	
	Men	Women	Men	Women
**Iron**	Cereals (18%)	Cereals (17%)	Cereals (19%)	Cereals (15%)
	Bread (12%)	Bread (15%)	Bread (14%)	Bread (15%)
	Beef and veal (8%)	Vegetables (8%)	Beef and veal (9%)	Vegetables (9%)
**Folate**	Cereals (20%)	Cereals (18%)	Cereals (19%)	Vegetables (16%)
	Vegetables (15%)	Bread (16%)	Bread (16%)	Bread (15%)
	Bread (14%)	Vegetables (16%)	Vegetables (14%)	Cereals (14%)
**Vitamin B_12_**	Milk (20%)	Milk (24%)	Milk (23%)	Milk (24%)
	Beef and veal (17%)	Fish and seafood (16%)	Beef and veal (21%)	Beef and veal (16%)
	Fish and seafood (16%)	Beef and veal (15%)	Fish and seafood (12%)	Fish and seafood (9%)

**Table 4:** Binary logistic regression, odds ratio of deficiency: Likelihood of being deficient in serum iron, serum vitamin B_12_ or RBC folate in Māori and non-Māori according to quartiles of dietary intake of iron (mg/day), vitamin B_12_ (µg/day) or folate (µg/day).

	Māori				Non-Māori			
**Ron Intake (mg/day)**	**Model 1 (Unadjusted)**		Model 2 (Adjusted) *		**Model 1 (Unadjusted)**		Model 2 (Adjusted) *	
	**(*n* = 8)**	***p***		***p***	**(*n* = 18)**	***p***		***p***
<7.75	1.00 (ref.)	-	1.00 (ref.)	-	1.00 (ref.)	-	1.00 (ref.)	-
7.75–10.42	0.43 (0.04, 4.39)	0.478	0.41 (0.04, 4.34)	0.458	0.63 (0.16, 2.45)	0.500	0.82 (0.19, 3.60)	0.789
10.43–13.27	0.75 (0.12, 4.80)	0.769	0.59 (0.08, 4.58)	0.617	0.29 (0.06, 1.58)	0.153	0.49 (0.07, 3.32)	0.465
>13.27	1.11 (0.17, 7.20)	0.912	0.76 (0.07, 8.41)	0.820	1.17 (0.35, 3.90)	0.802	2.33 (0.46, 11.9)	0.311
**Folate Intake (µg/day)**	**Model 1 (Unadjusted)**		**Model 2 (Adjusted) ¥**		**Model 1 (Unadjusted)**		**Model 2 (Adjusted) ¥**	
	**(*n* = 58)**	***p***		***p***	**(*n* = 111)**	***p***		***p***
<215	1.00 (ref.)	-	1.00 (ref.)	-	1.00 (ref.)	-	1.00 (ref.)	-
215–304	0.16 (0.06, 0.47)	0.001	0.16 (0.05, 0.47)	0.001	0.91 (0.45, 1.81)	0.781	0.90 (0.44, 1.86)	0.774
305–440	0.19 (0.06, 0.55)	0.002	0.13 (0.04, 0.43)	0.001	0.61 (0.31, 1.23)	0.166	0.60 (0.27, 1.31)	0.201
>440	0.10 (0.03, 0.32)	<0.001	0.06 (0.01, 0.34)	0.001	0.45 (0.22, 0.91)	0.027	0.59 (0.20, 1.69)	0.325
**Vitamin B_12_ Intake (µg/day)**	**Model 1 (Unadjusted)**		**Model 2 (Adjusted) ◊**		**Model 1 (Unadjusted)**		**Model 2 (Adjusted) ◊**	
	**(*n* = 15)**	***p***		***p***	**(*n* = 34)**	***p***		***p***
<2.07	1.00 (ref.)	-	1.00 (ref.)	-	1.00 (ref.)	-	1.00 (ref)	-
2.07–3.03	0.46 (0.08, 2.60)	0.378	0.39 (0.07, 2.28)	0.293	1.61 (0.56, 4.67)	0.379	1.41 (0.47, 4.21)	0.536
3.04–4.24	0.77 (0.18, 3.21)	0.717	0.57 (0.12, 2.65)	0.472	1.70 (0.58, 5.03)	0.338	1.33 (0.41, 4.29)	0.639
>4.24	0.62 (0.15, 2.56)	0.508	0.41 (0.08, 2.14)	0.293	1.21 (0.38, 3.87)	0.743	0.89 (0.24, 3.31)	0.862

Values expressed as odds ratio (95% confidence interval). *p*-value < 0.05 considered to be significant. Deficient serum iron concentration considered to be <10 µmol/L; Deficient red blood cell (RBC) folate concentration considered to be < 317 µg; Deficient serum vitamin B_12_ concentration considered to be <148 µg. * Model 2 adjusted for sex, age, energy intake and nutritional supplement intake. Model 2 adjusted for sex, age, energy intake, nutritional supplement usage and intake of folic acid. ◊ Model 2 adjusted for sex, age, energy intake, and intake of proton pump inhibitors, histamine-2 receptor antagonists, antacids, and nutritional supplements.
